# Historical Perspective of Traditional Indigenous Medical Practices: The Current Renaissance and Conservation of Herbal Resources

**DOI:** 10.1155/2014/525340

**Published:** 2014-04-27

**Authors:** Si-Yuan Pan, Gerhard Litscher, Si-Hua Gao, Shu-Feng Zhou, Zhi-Ling Yu, Hou-Qi Chen, Shuo-Feng Zhang, Min-Ke Tang, Jian-Ning Sun, Kam-Ming Ko

**Affiliations:** ^1^School of Chinese Medicine, Beijing University of Chinese Medicine, Beijing 100102, China; ^2^Research Unit for Complementary and Integrative Laser Medicine, Research Unit of Biomedical Engineering in Anesthesia and Intensive Care Medicine and TCM Research Center Graz, Medical University of Graz, Auenbruggerplatz 29, 8036 Graz, Austria; ^3^School of Basic Medicine, Beijing University of Chinese Medicine, Beijing 100102, China; ^4^College of Pharmacy, University of South Florida, Tampa, FL 33612, USA; ^5^School of Chinese Medicine, Hong Kong Baptist University, Hong Kong; ^6^American Academy of Natural Medicine, Costa Mesa, CA 92627, USA; ^7^Division of Life Science, Hong Kong University of Science & Technology, Hong Kong

## Abstract

In recent years, increasing numbers of people have been choosing herbal medicines or products to improve their health conditions, either alone or in combination with others. Herbs are staging a comeback and herbal “renaissance” occurs all over the world. According to the World Health Organization, 75% of the world's populations are using herbs for basic healthcare needs. Since the dawn of mankind, in fact, the use of herbs/plants has offered an effective medicine for the treatment of illnesses. Moreover, many conventional/pharmaceutical drugs are derived directly from both nature and traditional remedies distributed around the world. Up to now, the practice of herbal medicine entails the use of more than 53,000 species, and a number of these are facing the threat of extinction due to overexploitation. This paper aims to provide a review of the history and *status quo* of Chinese, Indian, and Arabic herbal medicines in terms of their significant contribution to the health promotion in present-day over-populated and aging societies. Attention will be focused on the depletion of plant resources on earth in meeting the increasing demand for herbs.

## 1. Introduction

Herbalism is a traditional medicinal or folk medicine practice based on the use of plants and plant extracts. Herbs/plants, the major component of traditional materia medica in the world, are of the main forms of life on earth. It is estimated that there are about 350,000 species of existing plants (including seed plants, bryophytes, and ferns), among which 287,655 species have been identified as of 2004 [1]. Herbal medicine (HM), also called botanical medicine, phytomedicine, or phytotherapy, refers to herbs, herbal materials, herbal preparations, and finished herbal products that contain parts of plants or other materials as active ingredients [2]. The plant parts used in herbal therapy include seeds, berries, roots, leaves, fruits, bark, flowers, or even the whole plants. Man was mainly dependent on crude botanical material for medical needs to retain vitality and cure diseases [3] prior to the introduction of aspirin derived from* Spiraea ulmaria* which was already prescribed for fever and swelling in Egyptian papyri and recommended by the Greek Hippocrates for pain and fever.

Although written records about medicinal plants dated back at least 5,000 years to the Sumerians, who described well-established medicinal uses for such plants as laurel, caraway, and thyme [4], archeological studies have shown that the practice of herbal medicine dates as far back as 60,000 years ago in Iraq and 8,000 years ago in China [5, 6]. With the advent of western medicine (or “conventional” medicine) over the past century, herbal medicine has been challenged by practitioners of mainstream medicine because of the lack of scientific evidence in the context of contemporary medicine, despite its long history of effective use. Interestingly, things change with time. In recent years, there has been a resurgence of the use of herbs due to the side effects of chemical drugs, lack of curative modern therapies for several chronic diseases, and microbial resistance, as well as the unprecedented investment in pharmaceutical research and development (R&D) [7]. For example, only about 1,200 new drugs have been approved by the US Food and Drug Administration (FDA) since 1950 [8]. As a result, the use of herbs and herbal products for health purposes has increased in popularity worldwide over the past 40 years, in both the developing and the industrialized countries [9]. Moreover, global pharmaceutical companies armed with modern science/technology and ideas have begun to rediscover herbs as a potential source of new drug candidates and renewed their strategies in favor of natural product drug development and discovery [10–13].

Nowadays, many practitioners of “conventional” medicine do not hesitate to recommend herbs, herbal products, or complementary and alternative medicine (CAM) therapy to their patients for the effective treatment of certain diseases [14, 15]. A survey in 2007 indicated that about 40% of adults and 11% of children used CAM therapy (CAMT), and among the adult users, white and black adults constituted 43.1% and 25.5%, respectively [16]. In addition, CAM and herbal medicines are more commonly used by people with higher levels of education and income [17, 18]. In this context, a 2012 study indicated that the use of CAM significantly correlated with higher education level, with a trend towards greater use in younger patients with breast cancer [19]. Although at present, we do not fully understand the exact facts and mechanisms underlying most traditional remedies and/or how they prevent disease that does not affect the enthusiasm of the public to accept CAM/CAMT [20]. Although there is a wide variety of CAM and CAMT around the world, they can all be divided into two main categories, namely, drug-based CAM/CAMT and non-drug-based CAM/CAMT [21].

Our earlier endeavors, which focused on discussing the current research and development of Chinese herbal medicine (CHM), and the trend in drug discovery, as well as a variety of CAM, aimed to promote the utilization of natural and traditional resources for contemporary health care, including food/diet therapy [7, 21–24]. As a continuing effort, the current paper will give an overview on herbal medicine from China, India, and Arabia, which are the three most influential traditional medicine systems to improve public health problems.

## 2. Chinese Herbal Medicine (CHM)

In ancient Chinese times “medicine” (traditional Chinese medicine, TCM, e.g.,* Zhong-Yi* in Chinese) and “pharmacy” (CHM, e.g.,* Zhong-Yao* in Chinese) were already described as distinct disciplines. More than 85% of Chinese materia medica (CMM) originates from plants, but animal parts/insects, minerals, and crude synthetic compounds are also prescribed by TCM practitioners. In addition, the term “CHM” also encompasses a number of ethnic herbal medicines and folk medicines in China.

### 2.1. Literature Overview of CHM

CHM is traditionally one of the most important modalities utilized in TCM. It has an extremely valuable, rich, lengthy, and extensive treatment history. CHM was firstly described by a legendary figure called* Shen-Nong*, who is said to have lived from 2737 BCE to 2697 BCE, nearly 5,000 years ago [25, 26]. It is said that* Shen-Nong, by* tasting hundreds of herbs on one day, found more than 70 herbs that had medicinal value, selected those that were suitable as remedies, and described their properties [27]. As a result of his efforts, numerous herbs (“herbal” medicine) became routinely used for health care in ancient China [28].* Shen-Nong-Ben-Cao-Jing*, the first known user guide to CHM, was written by authors who lived during the period immediately following the fall of the* Han* dynasty (202 BCE–220 CE). The compendium documented 365 Chinese herbal preparations, including 252 kinds of plant parts, 67 kinds of animal parts, and 46 kinds of minerals for medication, and it also described their therapeutic effects.

Prior to the time of* Shen-Nong-Ben-Cao-Jing*, some ancient Chinese scripts, such as* Shang-Shu*,* Shi-Jing* (The book of songs),* Shan-Hai-Jing*,* Zhou-Li*,* Li-Ji* (The book of rites), and* Zuo-Zhuan*, recorded the use of herbal remedies.* Shi-Jing*, which first recorded the use of herbal remedies, illustrated not only the therapeutic effects of the herbs, but also the places where the herbs were grown and their harvesting season. It recorded 170 kinds of CHMs, including 80 plant species and 90 insect species [29].* Shan-Hai-Jing*, the oldest Chinese book dealing with geography, recorded 9 species of plants with food value, 45 species of plants with medicinal value, 6 plants with some kind of efficacy, 6 plants poisonous to animals and pests, 6 species of plants with mood-elevating effects, 6 plants with health-promoting properties, 19 species of plants for the treatment of diseases, and 2 plants that are poisonous for humans [30].

More than 240 herbal drugs and 52 prescriptions were described in the book named* 52 Bing-Fang* (Recipes for 52 Ailments), which was unearthed in an ancient tomb (*Ma-Wang-Dui*) in China [31].* Xin-Xiu-Ben-Cao* (Newly revised materia medica), which was promulgated in 659 CE and recorded 850 kinds of herbal drugs, was the first pharmacopoeia in China, even in the world [32]. Oracle bone, a form of divination in ancient China, recorded more than 60 kinds of plants and animals, but they were not described as medication.

The* epic* book of materia medica in TCM history,* Ben-Cao-Gang-Mu* (*Compendium of Materia Medica*) written by* Li Shi-Zhen* (1528–1593), was published in 1596 in China. This book recorded 1,892 kinds of herbal medicines and 11,096 herbal formulae. After Charles Darwin (1809–1882) had read the book, he stated that* The Compendium of Materia Medica* was the encyclopedia of 16th century in China. This book was later translated into different languages, including Japanese, Korean, English, French, Russian, and Latin, and it has become a major historical reference on CMM.

The founding of China has brought about a hitherto unprecedented development of CHM in Chinese history. The holistic and systematic development of CHM has resulted in an increase in the number of approved CHMs.* Zhong-Hua-Ben-Cao*, the most authoritative Chinese book with a complete record of CMM issued in 1999, lists 8,980 kinds of CHMs that are divided into 34 volumes and summarizes the contemporary research of Chinese medicine with modern science and technology.* Zhong-Yao-Da-Ci-Dian* (a dictionary of traditional Chinese medicine), published in 1997, recorded 5,767 CHMs; when it was reprinted in 2006, the number of CHMs had increased to 6,008. The Chinese Pharmacopoeia (2010 version) listed 2,165 CHMs and their products. About 300 of them are commonly used in clinical practice, and many others are used locally as folk medicines. In terms of the literature on CHM, the theoretical aspects and practical experiences of several thousand years of usage are documented in more than 8,000 books; the total number of ancient literature about both CHM and TCM reached 13,000. Therefore, the documentation of knowledge in CHM is unique in the world (Table 1).

### 2.2. The Contribution of CHM to the World's Pharmacy

CHM has been influencing the world since ancient times. The famous Italian traveler Marco Polo (1254–1324) described a scene of merchants shipping Chinese herbs in Aden and Alexandria in his* Travel Book*. During the sea voyage of* Zheng He* between 1405 and 1433, China exported a large number of herbs including rhubarb, angelica, velvet, poria, taurine, ginseng, and cinnamon to other Southeast Asian countries. In return, over the past 2,000 years, more than 40 kinds of foreign herbs were imported into China and eventually adopted by TCM; they include kelp from Korea, turmeric and styrax from Southeast Asia, and others such as borneol, clove, frankincense, myrrh, benzoin, senna, and saffron [35].

In the 18th century, with reference to Chinese ginseng,* Panax quinquefolium* (also called American ginseng) was first discovered. It is indigenous to the southern regions of Ontario and Quebec in Canada and the midwestern, southern, and eastern parts of the United States [36]. In recent decades, studies have shown that American ginseng, like the Chinese one, also possesses neuroprotective, cardioprotective, antidiabetic, antioxidant, and anticancer properties, as well as the ability to alleviate symptoms of the common cold [37, 38]. The proven similarities between American ginseng and Chinese ginseng have been instrumental in boosting the market of the American product. From 1960 to 1992, both the demand and the price for American ginseng increased, with the export value being over US $104 million in 1992 in the USA alone. During the period from 1997 to 2007, the average export price of cultivated ginseng from the USA was US $19.30/lb and that of wild ginseng was US $84.50/lb [39]. The Panax family consists of at least nine species, including* ginseng*,* panax quinquefolium*, p*anax notoginseng* (*Sanqi*), and* Panax japonicus* (Japanese ginseng) [40]. If Chinese ginseng had not served as a reference herb, the Panax family would certainly not have become so popular and might still be treated as ordinary grass. Similarly, if the Chinese had not recognized the medicinal value of bezoars (gallstones from cattle), they would only have been treated as waste. Currently, China imports more than 100,000 kilograms of gallstones each year, about 60% of which comes from Africa, and the total value amounts to US $100 million. A good quality gallstone sells for between US $15 and 20 per gram [41].

In the winter of 753,* Jian Zhen* (688–763), a famous Chinese master of Buddhism in the* Tang* Dynasty, arrived in Nara, Japan, after several unsuccessful attempts, and he brought with him a number of CHMs. To date, more than 60 kinds of CHMs are still kept in the Nara Shosoin. According to the official body of Japanese kampo medicine (the practice of CHM in Japan), 36 kinds of CHMs were brought by* Jian Zhen* for use in Japan; they include ephedra, asarum, peony, monkshood, polygalaceae, astragalus, licorice root, angelica, bupleurum, Chuanxiong, scrophulariaceae, scutellaria, platycodon, anemarrhena, pinellia, schisandra, and eucommia [42]. To recognize the contribution of* Jian Zhen*, he was renowned as the father of kampo medicine by the Japanese. The 14th edition of the* Japanese Pharmacopoeia* (JP), issued in 1993, listed 165 herbal ingredients, the majority of Chinese origin, that are approved to be used in kampo remedies [43]; the 16th* Japanese Pharmacopoeia*, published in 2012, listed 276 kinds of crude drugs (e.g., herbal medicines and/or their extractions) [44].

All in all, the development of CHM has emerged from thousands of years of Chinese civilization. It is therefore no surprise that CHM is of great worth for mankind.

### 2.3. Species in China and CHM

**Figure 1:**
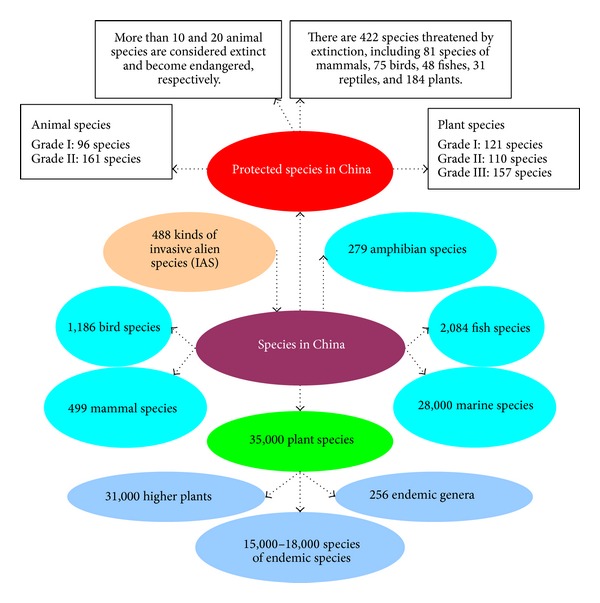
Species and protected species in China.

Nature has endowed China with a vast landscape with varied geographical features and a resultant wealth of medicinal plants. Geographically, China (from south to north) covers equatorial, tropical, subtropical, warm-temperate, temperate, and cold-temperate zones. Therefore, Chinese climatic conditions are suitable for the growth and reproduction of various animals and plants. In China, there are 499 kinds of mammals, 1,186 kinds of birds, 376 kinds of reptiles, 279 kinds of amphibians, and 2,084 kinds of fish, which account for 12.5, 13.1, 6.0, 7.0, and 12.1% of their respective species in the world [45–50]. China has more than 31,000 higher plants, 256 endemic genera, and 15,000–18,000 endemic species (50–60% of the total on earth), many of which are living fossils, such as dawn redwood (*Metasequoia glyptostroboides* Hu and Cheng), ginkgo (*Ginkgo biloba* L.), silver fir (*Cathaya argyrophylla* Chun and kuang), and tulip tree (*Liriodendron chinense* (Hemsl.) Sarg.) [51]. The increasing demand for herbal products in the global market is likely to challenge herbal resources in the world. In the* China Plant Red Data Book* published in 1992, 388 species of plants are listed as threatened, which include 121 as endangered (i.e., first grade national protection), 110 as rare (second grade national protection), and 157 as vulnerable (third grade national protection). Among these plant species, 77 are typical CHMs that account for 19.86% of the total threatened species [52]. Besides, 257 kinds of animal medicine appear in the national key protection name list of wild animals (Figure 1).

**Figure 2:**
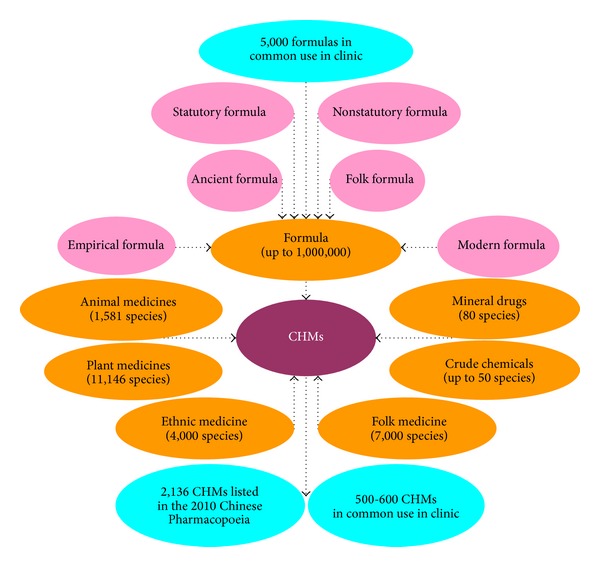
Chinese herbal medicine (CHM) in China.

In CHM, there are 11,146 different kinds of plants, 1,581 kinds of animals/animal parts and insects, 80 kinds of mineral drugs, and more than 50 kinds of crude chemical preparations, as well as 5,000 (total one million) clinically validated herbal formulations. Unlike other herbal medicines and western medicines, CHMs are often prescribed as formulas under the guidance of TCM's theories and practice. Each herbal medicine prescription (formula,* Fang-Ji* in Chinese) is a cocktail of many herbs tailored to the individual patient. It allows us to blend herbs to enhance their positive effects and reduce or eliminate any negative side effects they may have, when they are used each alone (Figure 2).

Because of the differences in geographical and climatic conditions, residents in various geographical regions in China have distinctive lifestyles, customs, and cultures, as well as disease spectra. These variations have brought about the development of a wide variety of traditional medicine practices. China has 56 ethnic groups, meaning that there are 56 kinds of culture, language, and herbal medicine. CHM (also called* Han* medicine) was developed by the* Han* ethnic group.  Table 2 shows the number of plant-derived herbal medicines in various ethnic medicines as recorded in the database of China plant species. In fact, the number of herbal medicines is far bigger than that recorded in the database. For example, Tibetan herbal drugs have 2,172 rather than 1,085 varieties, not including 214 kinds of animal drugs and 50 kinds of mineral drugs [53]. There is no doubt that CHM, together with other ethnic herbal medicines in China, comprises a gold mine of potential modern medicines and health products.

### 2.4. Pharmaceutics of CHM

Dosage form, also known as routes of administration, is a mixture of components with medicinal properties and nondrug components (excipient or vehicle). It describes the physical form in which medication will be delivered into the body. Currently, there are more than 40 available dosage forms of CHM products in the market. They include decoction (*Tang-Ji*, hot water extract), tincture (*Ting-Ji*, ethanol extract), powders (*San-Ji,* powder form), bolus/pills (*Wan-Ji*, boluses or small pills containing herbal ingredients), pastes (*Jin-Gao*, extracts from organic solvents), granules, tablets, oral liquids, and injection liquids. Liniment, poultices, plasters, and ointments are adopted for external use of CHM. Recently “nanomized” and “aerosol” herbs have emerged as new dosage forms of CHM [54–56].

**Figure 3:**
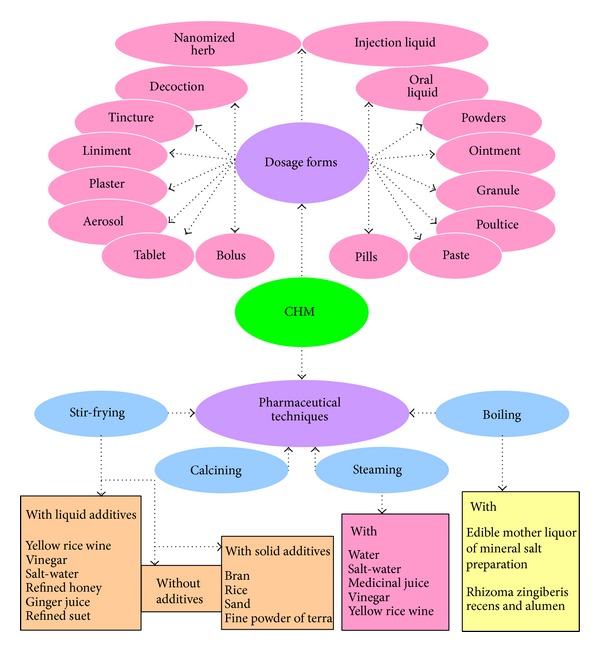
Dosage forms and pharmaceutical techniques in Chinese herbal medicine (CHM).

Unlike synthetic drugs, CHMs are usually subjected to specific treatments (processing, the process of preparing CHM), also known as* Pao-Zhi* in Chinese, prior to use.* Pao-Zhi* is a very ancient part of the practice of Chinese medicine, dating back at least 2,000 years. There are more than 30 kinds of procedures involving stir-frying (*Chao*), calcining (*Daun*), steaming (*Zheng*), boiling (*Zhu*), and so forth. The herbal effects and compositions/ingredient structures are changed after* Pao-Zhi*, as compared to the unprocessed version. Experience has shown that the effectiveness and security of some CHMs are dependent upon their correct* Pao-Zhi* before being used in clinic. This is one of the reasons why CHM is different from the plant drug and/or natural drug. Regardless of the primitive processing technology that was used in ancient times, the rationale underlying the traditional processing of CHM has been supported by scientific evidence in modern research.  Figure 3 shows the traditional processing methods of CHM, together with their pharmaceutical processing procedures, which are still employed in the pharmaceutical industry of CMM in China [57].

### 2.5. The Status Quo of CHM

The CHM industry has always been one of China's traditional competitive industries. In 2010, CHM manufacturing assets in China exceeded 300 billion RMB, an increase of about 18%, nearly 5 percentage points higher than the year before; the number of CMM pharmaceutical enterprises amounts to more than 2,300, total investment in fixed assets totaled nearly 500 billion RMB, an increase of about 16% compared to the previous year [58]. As of today, there are 8,000 products related to CHM in the China market. In 2010, China manufactured 2.384 million tons of Chinese herbal products, with sales amounting to 417.875 billion RMB [59].

More than 8,000 varieties of CHMs or related herbal products are now exported from China to more than 130 countries and regions worldwide; each year, more than 50 kind of CMMs are exported to the United States, including berberine, angelica, licorice, Fritillaria, turmeric, frankincense, Tianma, rhubarb, Eucommia, cloves, wolfberry, Panax, fresh ginseng, and pinellia [60]. Over the past few years, herbal exports have steadily increased from US $1.09 billion in 2006 to US $1.46 billion in 2009 [61].

**Figure 4:**
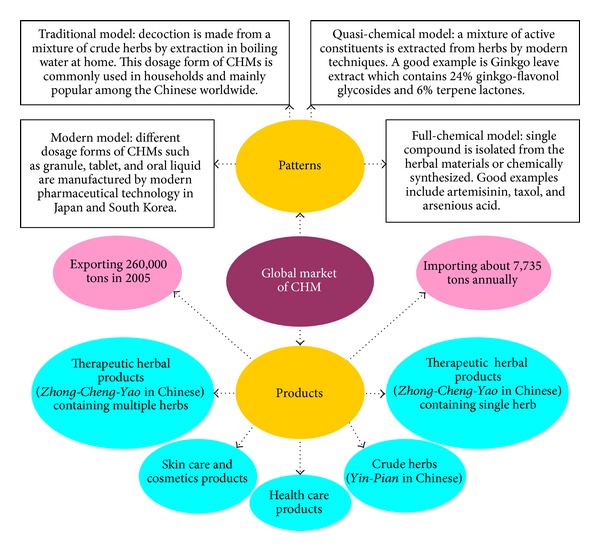
Styles of Chinese herbal medicine (CHM) in the global market (see also [62] of quasi-chemical model and [63–65] of full-chemical model).

More importantly, in recent decades, China has put a great deal of human efforts and financial resources to promote research and development in the area of CHM in a systematic manner, and this enormous effort is unmatched by other traditional medicines around the world. In this context, we have published reviews on the status of CHM research and development as well as drug discovery in China [24]. In China, 3,563 extracts, 64,715 compositions, 5,000 single compounds, and 130 kinds of CHM-related chemical drugs have been developed [22]. From the marketing perspective, currently, four models of application and five types of Chinese herbal products can be adopted in the international arena. The same approach may also be applied to other herbal medicines (Figure 4).

## 3. Indian Herbal Medicine (IHM)

Indian medicine/materia medica/herbal medicine (IM/IMM/IHM), also called Ayurvedic medicine/materia medica (AYM/AYMM), belongs to the traditional health care and longevity systems. Because the belief that “everything can be a drug” is deeply rooted in Indian culture, Ayurvedic physicians made use of an extensive collection of medications, herbs/plants, even the urine of animals, and described their effects meticulously. Currently, 70 percent of Indians still rely on IM for their primary health care [66].

### 3.1. Literature Overview of IHM

In India, the history of using plant resources for treating diseases can be dated back to 6,000 to 4,000 BCE, the Buddhist period. AYM has a vast literature in Sanskrit and various Indian languages, covering various aspects of diseases, therapeutics, and pharmacy. The earliest references to such plants, minerals, and animal products with their usage for medical purposes are found in the* Rig veda*, an ancient Indian sacred collection of Vedic Sanskrit hymns, and the* Atharvaveda*, the fourth and last Veda of Hindu literature [67].* Bhava Prakasha*, written by Bhava-Mishra, is the most important text on herbs/plants and is held in high esteem by modern Ayurvedic practitioners [68–70].

**Figure 5:**
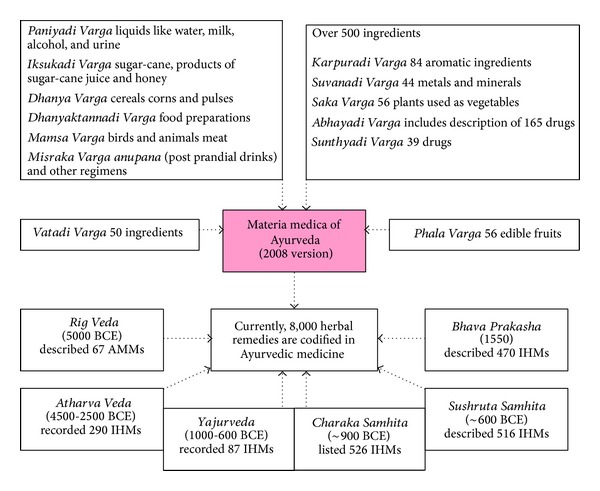
Some important texts of Indian herbal medicine [76–78].

The oldest text of AMM, the* Rasa Vaisesika* of Nagarjuna, who is considered the most important Buddhist philosopher after Buddha's death [71], was composed during the 5th century CE. In this text the various concepts of drug composition and action were described [72]. The* Charaka Samhita* is the first recorded treatise fully devoted to the concepts and practice of Ayurveda, with a primary focus on therapeutics [73]. In the* Charaka Samhita*, plant-derived drugs are divided into 50 groups according to their pharmacologic/therapeutic actions. The next landmark in Ayurvedic literature was the* Sushruta Samhita*. Although the text places special emphasis on surgery, it also describes 395 medicinal plants, 57 drugs of animal origin, and 64 minerals or metals as therapeutic agents [74] (Figure 5). In ancient times, Ayurvedic texts were very respected in the neighboring countries, and they were also translated into Greek (300 BCE), Tibetan and Chinese (300 CE), Persian and Arabic (700 CE), and so forth [75].

### 3.2. Plant Species in India and IHM

India possesses almost 8% of the estimated biodiversity of the world with around 126,000 species; there are about 400 families of flowering plants in the world, at least 315 of these can be found in India [79]. Currently, about 45,000 species (nearly 20% of the global species) are found in the Indian subcontinent: ~3,500 species of plants are of medicinal value; 500 medicinal plant species are used by the contemporary Ayurvedic industry; ~80% of the medicinal plant species are procured from wild areas; and 10% of medicinal plants involved in active trade are obtained from cultivation in farms [80]. The western Himalayan region provides about 80% of herbal drugs in Ayurveda, 46% of Unani, and 33% of allopathic systems [81]; 50% of drugs recorded in the British Pharmacopoeia are related to medicinal plants growing in this region [82].

In India, approximately 25,000 plant-based formulations are used in traditional and folk medicines [83]. The number of plant species used in various IM is as follows: Ayurveda, 2,000; Siddha (a type of ancient traditional Indian medicine), 1,300; Unani (a system of alternative medicine first developed by the Islamic physician Avicenna in about 1025 CE), 1,000; homeopathy, 800; Tibetan, 500; modern, 200, and folk, 4,500 [84]. More than 7,500 plant species are currently used in IM, including tonics, antimalarials, antipyretics, aphrodisiacs, expectorants, hepatoprotectants, antirheumatics, and diuretics [85, 86], as well as for the therapy of certain central nervous system disorders [87, 88].

The IHMs are derived either from the whole plant or from different organs, like leaves, stem, bark, root, flower, seed, and so forth, but also include animals and minerals. Some drugs are prepared from excretory plant products such as gum, resins, and latex. Commonly used spices, herbs, and herbal formulae are utilized for therapeutic interventions in about 28 kinds of chronic diseases in humans [89]. Special herbal preparations, known as* Rasayans*, are used for rejuvenation and retarding the aging process, thereby promoting longevity [90].

**Figure 6:**
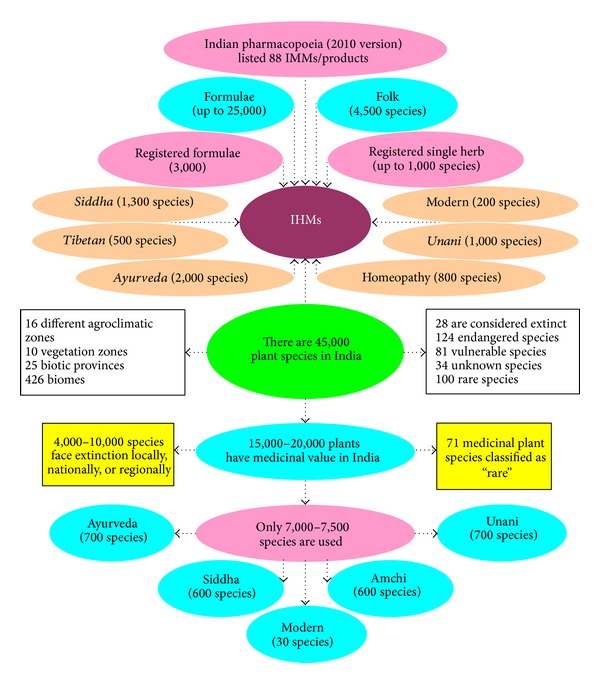
Plant species in India and Indian herbal medicine (IHM).

Of the 700 plant species commonly used in the Indian herbal industry, 90% of them are collected from the wild. About 50% of the tropical forests, the treasure house of plant, and animal diversity have already been destroyed. Many valuable medicinal plants are on the verge of extinction. The* Red Data Book of India* in 1997 has 427 entries of endangered species of which 28 are considered extinct, 124 endangered, 81 vulnerable, 100 rare, and 34 insufficiently known species [91]. The* Red Data Book of India* released in 2012 described 3,947 species as “critically endangered”, 5,766 as “endangered”, and more than 10,000 species as “vulnerable” [92] (Figure 6).

### 3.3. Pharmaceutics of IHM

Compared to those of CMM, AYM possesses very complex formulae consisting of 30 or more ingredients. In the formula, a number of ingredients, which are properly processed for pharmaceutical application, are chosen to balance the three humoral doctrines (“*Vata*”, “*Pitta*,” and “*Kapha*”). Herbs used in AYM include essential oils extracted from plants, fruits, vegetables, and common spices. The crude herbal material may be ground into powders and put into capsules, cooked into teas, used topically, taken raw, and so forth. IMM preparations on the market and/or Ayurvedic medical practice are complex mixtures including plant- and animal-derived products, minerals, and metals, as well as involving several specific preparatory steps or manufacturing processes.

*Kasthoushadhies* (herbal preparations) and* Rasaoushadhies* (herbo-bio-mineral-metallic preparations) are the two major groups of IMM preparations [93]. The latter has a metallic base but ordinarily does not contain active metal, since the metal is converted into an ash or oxide and forms an organometallic compound with a number of organic materials used for trituration as* Bhavana Dravya *[94, 95].

Medicinal principles are present in different parts of the plant such as root, stem, bark, heartwood, leaf, flower, fruit, or plant exudates. Generally, the herbal remedies can be in various crude dosage forms like pills, powders, essential oil, infusions, or poultices. AYM believes that* Sandhana kalpana* (biomedical fermented formulations), a unique and complex dosage form containing acidic and alcoholic fermented components, is one of the most effective dosage forms of Ayurveda in practice for thousands of years [96]. During the fermentation process of liquid basic drugs, such as juices or decoctions, alcohol is produced by in-source material used in pharmaceutical procedure. Thus, extraction of active principles of the herbal drugs is done through self-generated alcohol. This formulation has longer shelf life, quick absorption and action, and excellent therapeutic efficacy as compared to other preparations [97].

**Figure 7:**
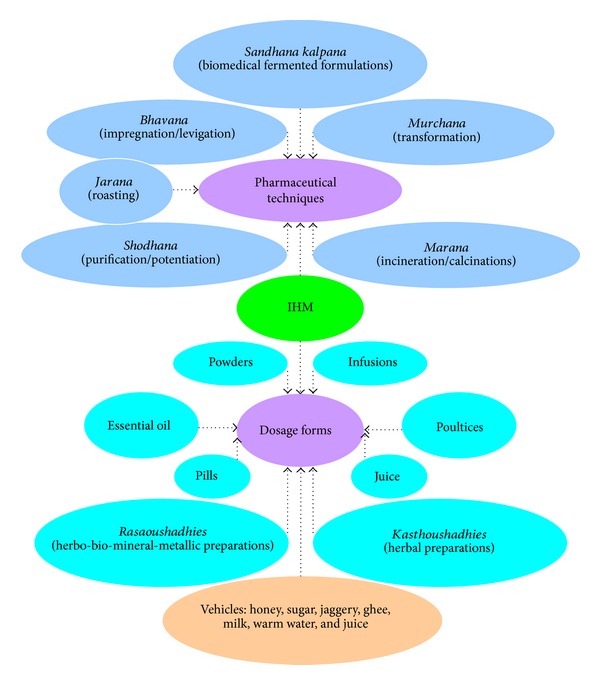
Dosage forms and pharmaceutical techniques in Indian herbal medicine (IHM) [99, 100].

The specific media are usually used in the manufacturing process of IMM products according to the different preparation. This plays a very important role in either breaking down the chemical compound(s) that is not required or forming the novel active ingredient(s) that is of value to the people, for example,* Shodhana* (purification/potentiation) of particular poisonous herbs, like* Gomutra* (cow's urine) for* Shodhana* of* Vatsanabha* (*Aconitum ferox* Wall.) and* Godugdha* (cow's milk) for* kupeelu* (*Strychnos nux-vomica Linn*.) [98]. On the other hand, Ayurvedic drugs are usually administered orally along with vehicle materials (*Anupana*) such as honey, sugar, jaggery, ghee, milk, warm water, and juice of some medicinal herbs. These Ayurvedic* Anupana* (i.e., drug vehicles serving as a medium of administration) can improve acceptability and palatability and help in absorption of the main herbal remedy; moreover, they may also act as an early antidote (Figure 7).

### 3.4. The Status Quo of IHM

The treatment of disease by Ayurveda is highly individualized and depends on the psychophysiologic status of the patient, particularly in relation to the season of the year [101]. Currently, more than 600 herbal formulas and 250 single plant drugs are included in the “Pharmacy” of Ayurvedic treatments [102]; about 1,000 single herbal remedies and 3,000 compound herbal formulations are registered in India. More than 600 herbal formulae and 250 single plants are included in the “Pharmacy” of Ayurvedic treatment [103]. The 6th* Indian Pharmacopeia* released in 2010 recognized 55 crude herbal drugs, 26 extracts, 3 finished formulations, and 2 pharmaceutical aids that are marketed [104].

According to a study commissioned by the Associated Chamber of Commerce and Industry, the Indian herbal industry is projected to double to 150 billion Rs. by 2015, from the current 75 billion business [105]. In the 1990s, the annual sales of the Indian herbal industry were about 23 billion Rs. (as compared to 145 billion Rs. in the pharmaceutical industry), with a growth rate of 15% [106]. By the end of 2012, the domestic market is expected to reach 145 billion Rs. and the export market 90 billion Rs., with compound annual growth rates of 20 and 25%, respectively [105]. The export market for medicinal plants appears to be growing faster than the Indian domestic market.

India not only has a great role to play as a supplier of herbal products for the domestic market, but it can also benefit from the tremendous potential afforded by overseas markets. Currently, the Indian herbal market is valued at 70 billion Rs., and over 36 billion Rs. worth of raw herbal materials and herbal products is exported [106]. The export of crude herbal extracts amounted to US $80 million, and the total sales of herbal products added up to US $1 billion [107]. Among the exported herbal products, 60% are processed plant materials that are unique to India, 30% are plant extracts, and 10% are Ayurvedic preparations [108]. The plant-derived pharmaceuticals exported from India include isabgol, opium alkaloids, senna derivatives, vinca extract, cinchona alkaloids, ipecac root alkaloids, solasodine, diosgenin/16DPA, menthol, gudmar herb, mehdi leaves, papian, rauwolfia guar gum, jasmine oil, agar wood oil, and sandal wood oil [109]. However, the export of 29 medicinal plants, including plant parts and their derivatives/extracts, obtained from wild sources, is prohibited by the Indian government [109]. Indian herbal medicine has now become a rich source of innovative drug discovery [110].

In India, the turnover of IHM industry is estimated to be more than 88 billion Rs; the domestic market is of the order of 40 billion Rs with a total consumption of all IMMs to a figure of 177,000 metric tons (MT). India has 9,493 HM manufacturing units, but 8,000 of them are small scale, one having an annual turnover of less than 10 million Rs. Some of the well-known units (with an annual turnover of more than 500 million Rs.) include Dabur, Zandu, Himalaya, Shree Baidyanath, and Arya Vaidya. They consume about 35% of the total raw IHMs [111].

## 4. Arabic Herbal Medicine (AHM)

It is well known that ancient Hippocratic-Greek medical knowhow was adapted and improved by Arabian herbalists, pharmacologists, chemists, and physicians in the Middle Ages. Furthermore, the majority of Arabs are Muslims, and Arabic culture and Islamic ideology are closely related. As such, Arabic medicine/materia medica/herbal medicine (AM/AMM/AHM) may also be called Greco-Arab or Islamic medicine.

### 4.1. Achievements of AM

The Arabic world used to be the center of scientific and medical knowledge for many centuries (from 632 to 1258 CE) after the fall of the Roman Empire.* The Legacy of Islam* (published in 1931; edited by the late Sir Thomas Arnold and Alfred Guillaume) states, “Looking back we may say that Islamic medicine and science reflected the light of the Hellenic sun, when its day had fled, and that they shone like a moon, illuminating the darkest night of the European Middle Ages; that some bright stars lent their own light, and that moon and stars alike faded at the dawn of a new day: the Renaissance. Since they had their share in the direction and introduction of that great movement, it may reasonably be claimed that they are with us yet.” [112].

During the middle ages, AM contributed greatly to the development of modern medicine and pharmacy in Europe. For instance, the European pharmacopoeia relied on Muslim writings and information therein until the late 19th century. Despite the scarcity of medical knowledge in the Koran, Arabs adopted the ancient medical practices that originated from Mesopotamia, Greece, Rome, Persia, and India (or even China) [113, 114]. In the early 11th century, Avicenna (980–1037), a great philosopher and physician, incorporated a number of Chinese herbal preparations in his book* Pharmacopoeia*. Ancient Arabs established their “Pharmacy” on the basis of physicochemical techniques such as evaporation, filtration, distillation, sublimation, and crystallization used in “alchemy” which was invented by the Chinese [115].

Alchemy is the predecessor of chemical discipline that led to the development of natural science in modern times. Therefore, China is regarded as one of the key players in advancing modern civilization, particularly in the area of scientific methodology. It has been stated that if Greece was the theoretical founder of modern civilization, the* Qin/Han* dynasty in ancient China was the technical founder of modern natural sciences. Alchemy was invented during the period of Warring States in ancient China, but it vanished for no apparent reason in the middle of the* Tang* Dynasty. Nevertheless, the spirit of exploration of the ancient Chinese is praise-worthy. Several inventions by Taoist alchemists, such as cinnabar (*Zhu-Sha*), orpiment (*Ci-Huang*), and realgar (*Xiong-Huang*) in CHM, particularly gunpowder, have had a far-reaching impact on modern medicine and on the world in general [115].

**Figure 8:**
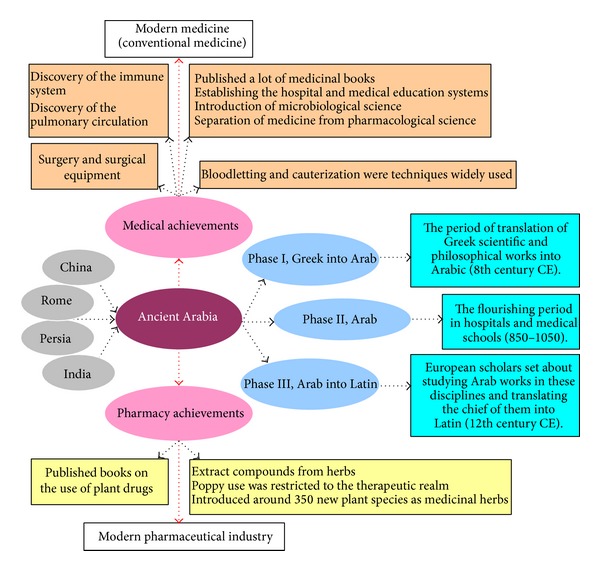
Achievements of Arabic medicine and pharmacy [118, 119].

Although AM was at the forefront of medical knowledge in Renaissance Europe of the 15th century, unlike CM and IM, its herbal medicine was not well developed from the start. The theory of AM is based on the “humours” of Hippocrates and Galen. There were more “modern” than “traditional” elements in AM; therefore, it played a pivotal role in the early formation and development of modern medicine. AM mainly integrated various herbal medicines and related technologies that originated from other countries and regions and established the foundation for the development of medicine and pharmacy in modern medicine [116, 117]. Therefore, AM carried on the past heritage and opened up the future in the history of the development of human medicine (Figure 8).

### 4.2. Past and Present of AHM

During the 8th century, Arabs in the Baghdad region were the first in history to separate medicine from pharmacological science. The world's first drug stores were established in the Arab world (Baghdad, 754 CE). The forms employed in that period are still used in therapy, and some formulations of drugs can be found in pharmacopoeias even today [120]. The earliest records of herbs, which were written on clay tablets in cuneiform, were from Mesopotamia (dating back to 2600 BCE). The best known Egyptian pharmaceutical record is the* Ebers* Papyrus (dating back to 1500 BCE), which documented some 700 herbal medicines (mostly from plants), with dosage forms including gargles, snuffs, poultices, infusions, pills, and ointments and vehicles using beer, milk, wine, and honey [121].

Since the 8th century, the practice of AHM has been using natural remedies, both organic (such as camel urine) and inorganic types, for the prevention and treatment of diseases [122]. Interestingly, pharmacological studies have revealed that camel urine treatment caused a significant cytotoxic effect on bone marrow cells in mice [123]. The Middle East region is inhabited by more than 2,600 plant species, of which more than 700 species are noted for their use as medicinal herbs or botanical pesticides; however, only 200–250 plant species are still in use in traditional Arab medicine for the treatment of various diseases [124]. Plant species from the western Mediterranean coastal region (from Alexandria to Sallum, Egypt) comprise 230 species belonging to 48 families; 89% of the species had medicinal value, 62% of the species were common, approximately 24.9% were occasional, and 13% were rare [118].

Until now, 236 plant species, 30 animal species, 29 organic substances, and 9 materials of other or mixed origins are still being used in treating human diseases and are sold or traded in the Mediterranean region and/or in the global market [124]. A survey of the plant species in the Mediterranean region by ethnopharmacologists indicated that 250–290 plant species are still in use [125, 126]. In Israel, 129 plant species are used in AM for the treatment of various diseases. Among these plants, there are 40 species used for treating skin diseases, 27 species for treating digestive disorders, 22 species for treating liver diseases, 16 species for treating respiratory diseases and coughing, 22 species for treating various forms of cancer, and 9 species for weight loss and lowering cholesterol [127]. However, more than 1,400 kinds of herbal medicines were used by Islamic physicians during the period of the Arab Empire (632–1258).

**Figure 9:**
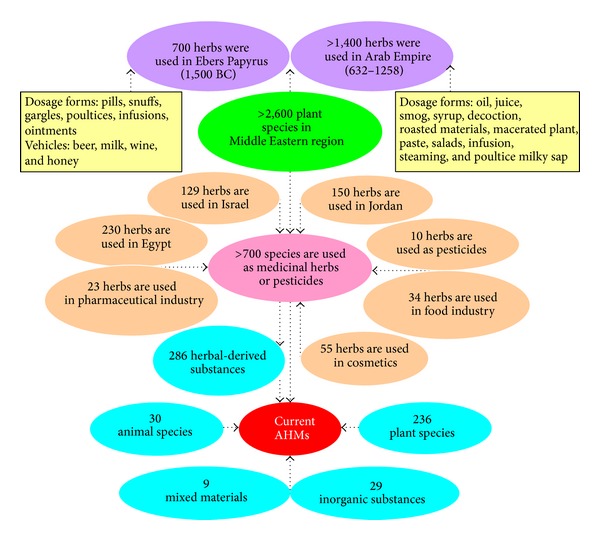
Past and present of Arabic herbal medicine [130].

The dosage forms utilized in AHM include decoction, infusion, oil, juice, syrup, roasted materials, fresh salads or fruits, macerated plant parts, milky sap, poultice, and paste, of which some formulations of herbal drugs are still used today [128]. Although AHM is the first choice for many people in dealing with ailments in the Middle East, most of the herbalists (such as those in Jordan), who acquire the expertise from their predecessors, are not properly trained in herbal medicine [129] (Figure 9).

In contrast to CHM or IHM, the physical characteristics of the herbal size, shape, color, texture, and taste traditionally served as important criteria in their selection for therapeutic purposes. For example, seeds with kidney shape are used for treating kidney stones; roots shaped similar to the human body or fruits that resemble human testicles are traditionally used to stimulate sexual desire or treat sexual weakness; a yellow decoction or juice obtained from herbal leaves is used for treating jaundice and liver diseases [131, 132].

## 5. Discussion 

In this section, two important issues related to herbal medicine are discussed.

### 5.1. The Theoretical Advantages of Herbal Medicine

Due to shortage of scientific evidence on the molecular mechanism of herbs, it is often considered as only an alternative choice to conventional drugs. Here, we attempt to describe the feasibility and superiority of herbal medicine containing complex and multicompounds as medication using logical concepts in philosophy.

Currently, multidrug therapy or polypharmacy, also known as multiple drug intake or cocktail treatment, which involves therapeutic interventions using combinations of drugs (herbal versus chemical, herbal versus herbal, and chemical versus chemical) through pharmacokinetic and pharmacodynamic pathways or both [133–136], is commonly practiced in clinical situations. It is believed that multidrug therapy produces beneficial effects that do not occur when using each drug alone. Due to the additive and/or synergistic interactions among the drugs, or the suppression of adverse effects, multidrug therapy appears to be effective in treating diseases such as cancer, AIDS, malaria, diabetes, hypertension, MRSA, and chronic diseases associated with old age. Nevertheless adverse drug reaction (ADR), another important public health problem, may be enhanced after multidrug combination treatment through not only drug-drug interaction, but also herb-herb or drug-herb interaction [137, 138]. For example, as a monotherapy, St John's wort extract has an encouraging safety profile. However, in some cases, life-threatening interactions were reported when used together with other drugs [139]. Therefore, the possibility of drug-drug interaction (DDI), including both beneficial effects and ADR, has caused the FDA and European Medicines Agency (EMA) to encourage the industry to perform drug interaction studies [140]. In the new postgenomic era DDI can be predicted with the data from pharmacogenetic information which may have an important implication for the development of personalized medicine and drug R&D for clinic and pharmaceutical industry, respectively [141].

More often than not, the pathogenesis of diseases is related to multiple targets rather than a single target. Asai et al. found that nonsteroidal anti-inflammatory drugs, cholesterol-lowering statins, and *β*- or *γ*-secretase inhibitors can produce additive effects on the reduction of A*β*-amyloid levels in cultured neuronal cells [142]. Combination therapy of PA-824-moxifloxacin and pyrazinamide can kill over 99% of drug-sensitive and multidrug-resistant* Mycobacterium tuberculosis* in patients with tuberculosis (TB) within 2 weeks. However, at present, the treatment of patients with TB or multidrug-resistant TB using conventional drug therapy requires 6 or 18–24 months, respectively [143]. A polypill containing amlodipine, losartan, hydrochlorothiazide, and simvastatin produces a significant effect in preventing heart attacks and strokes [144].* Liu Wei Di Huang Wan* (Rehmannia Six Formula), which is a well-known Chinese herbal formula used for the treatment of 137 kinds of diseases in China, consists of six Chinese herbs:* Radix Rehmanniae* nourishes kidney* Yin* and* essence* (minute substances for supporting life);* Fructus Corni* nourishes the liver/kidney and restrains the leakage of the* essence*;* Rhizoma Dioscoreae* tonifies spleen* Yin* and consolidates the essence;* Rhizoma Alismatis* promotes urination to prevent buildup of significant fluids;* Poria* drains dampness from the spleen;* Cortex Paeoniae* clears liver fire [145–147]. Therefore, the multitarget herbal formula can produce a wide range of therapeutic effects.

Herbal formulations evolved from thousands of years of experience in practicing herbal medicine. While therapeutic interventions using multiple drugs in modern medicine are based on an understanding of disease processes and drug mechanisms, the use of multicomponent herbal formulae (*Fu-Fang* in Chinese herbal medicine) is based on CM theory and practical experience. Unlike using a single drug in orthodox medicine, raw plant or plant extracts contain an array of bioactive ingredients (a single plant contains 100–1,000 compounds of 20–50 different structure types) that can produce additive and/or synergistic actions [148]. The multi-ingredient herbal drug/formula allows for a multitarget interaction in treating diseases. For instance, the common cold is a viral infectious disease of the upper respiratory system, which primarily affects the nasal cavity. However, cold symptoms typically include coughing, sore throat, runny nose, headache, fever, and discomfort in the entire body. So far, no single chemical entity can simultaneously alleviate all clinical manifestations of common cold. Therefore a typical over-the-counter cold remedy is composed of multiple drugs, such as aspirin (A), phenacetin (P), and caffeine (C), in an APC tablet.

**Figure 10:**
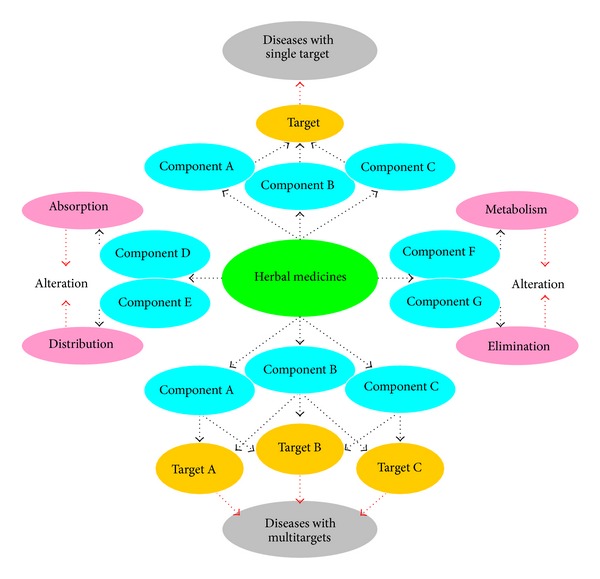
Pharmacodynamic synergism and pharmacokinetic synergism of herbal medicines.

One and one can add up to more than two. Therefore, herbal treatment resembles a cocktail treatment or “magic shrapnel” (multidrugs act on multiple targets) [149]. The chemical compounds residing in an herbal drug or formulation work together within the body to maintain health and/or fight against diseases. The concept of synergism in modern pharmacology encompasses two aspects: (1) pharmacodynamic synergy results from the enhancement of action when multiple biologically active substances are directed at related targets in a physiological system, which are often linked to the pathogenesis of a disease and (2) pharmacokinetic synergy can result from alterations in drug absorption, distribution, metabolism, and/or elimination (Figure 10).

### 5.2. Resource Conservation in Herbal Medicine

Excessive medical treatment and medication, including the consumption of herbal medications, is a global trend, especially in developed countries. Countless facts have indicated that herbal preparations or formulations can be used for the treatment of many common as well as complex diseases for all ages, with a minimum of adverse side effects compared to conventional drugs. Together with the long history of their use, plant-derived herbs and herbal products are gaining popularity in the global market as registered drugs, dietary supplements, health care products, cosmetics, and so forth. Medicinal plants are highly esteemed as a rich source of new therapeutic agents for the prevention and treatment of diseases. Nowadays, the public acceptance of herbal medicine increases not only in Asian countries (49% in Japan, 45% in Singapore, 70% in China, and 80% in India), but also in western countries [150]. The sales of herbal drugs or related products are expected to increase at an annual rate of 6.4%. In the USA, the use of herbal products by consumers was less than 5% in 1991, but it increased to 50% in 2004, and the amount of botanical remedies constitutes as much as 25% of total medications. According to a WHO report, the global market value of herbal products to date is US $61 billion, but it is predicted to grow to US $5 trillion by 2050 [151]. The market shares in Europe and the USA are 41 and 20%, respectively [152].

Of the 250,000 higher plant species on earth, more than 80,000 are of medicinal value even in the genome era. In Brazil, it is estimated that there are almost 55,000 native species, at least 1,200 documented medicinal plants, and probably many more undocumented species used by various indigenous groups [153]. It can be expected that natural medicines, particularly herbal medicine, will make a growing or even a decisive contribution to human health care again. By 2001, researchers had identified 122 compounds used in modern medicine which were derived from plant/herb sources. Of these, 80% have an ethnic medical use which is identical or related to the current use of the active component(s) of the plant [154]. Some of these compounds include tubocurarine, morphine, codeine, aspirin, atropine, pilocarpine, ephedrine, vinblastine, vincristine, taxol, podophyllotoxin, camptothecin, digitoxigenin, gitoxigenin, digoxigenin, capsaicin, allicin, curcumin, and artemisinin. Unfortunately, many plant species on earth have become endangered as the consumption of herbs and herbal products continues to increase world-wide.

Traditional herbal medicine uses remedies derived from plants, animals, metals, and minerals. If herbal resources are inappropriately exploited, the extinction of many plant species will inevitably occur, with a resulting adverse alteration of the ecological environment. For example, for more than 30 years wild* Panax notoginseng* has no longer been found in Yunnan province (the origin of the plant) in China [155]; the acquisition of one kilogram of wild licorice will destroy 8–10 acres of grasslands [156]; digging of one Cordyceps can cause direct damage to about 30 cm^2^ of grassland [157]. To treasure and maintain the gifts from mother nature (Hindu philosophy regards the Earth as a living being, i.e., mother nature), governments should install measures to ensure the ethical exploitation of herbal resources in their countries or societies. Therefore, it is high time to formulate strategies to avoid the overexploitation of herbal resources.

## 6. Concluding Remarks

Since ancient times, disease has been a leading cause of morbidity/mortality, and it is associated with a heavy economic burden among people with diseases. Despite current advances in science and medicine, disease remains a serious threat to public health in both developed and developing countries, urban and rural areas, and all ethnic groups. Ancient and modern people take medicines to fight illness or to feel better when they are sick. Most medicines (conventional drugs) at present are chemically synthesized and some are isolated from naturally occurring plants on the basis of their use in traditional medicine. However, our ancestors took only certain kinds of specific natural remedies to fight or prevent a specific illness. Because modern drug development is a high-risk (and therefore high-failure) commercial endeavor and synthetic drugs have a high rate of adverse events; there is a universal trend of using herbal medications or related products.

**Figure 11:**
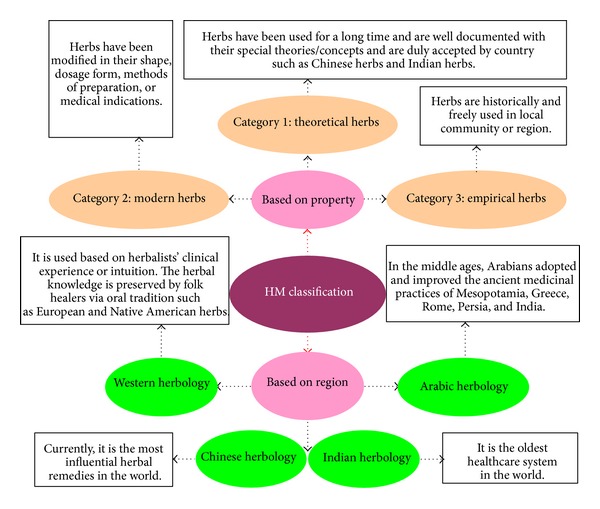
Classification of herbal medicines (HMs) in the international market.

Based on cultures and geographical regions, various kinds of herbal remedies have evolved. Herbal medicines are therefore an integral part of culture and geographical environment, and various kinds of herbal medicines have their own unique way of understanding and treating a disease. However, the globalization of trade and market has brought about an integration of different kinds of herbal medicines over the world. At present, herbal medications or related products in the global market are derived from Chinese herbs, Indian herbs, Arabic herbs, and Western herbs. Herbal remedies may also be classified into three categories, namely, modern herbs, theoretical herbs, and empirical herbs, in accordance with their nature/characteristics and the nature of current usage [158]. As for the medications derived from herbs, they no longer belong to any herbal series or category and have essentially become equivalent to conventional drugs. In general, most herbal remedies/formulae are considered to be safe and are well tolerated because they have been successfully used for thousands of years as foods to promote health and as medicines to treat diseases. To date, herbal products are widely available to consumers and have become increasingly popular throughout the world. There is no doubt that herbal products will continue to play a crucial role in the health care system of human societies, not to mention that secondary metabolites of plants are economically important as drugs, fragrances, pigments, food additives, and pesticides (Figure 11) [159].

## Figures and Tables

**Table 1:** Some important texts in the historical developmental process of Chinese materia medica [33, 34].

Lectures	Issued date	Total	Plant	Animal	Mineral	Processing products	Formulae	Other
*52 Bing-Fang *	200 BCE	247	115	48	21			63
*Shen-Nong-Ben-Cao-Jing *	202 BCE–220	365	252	67	46			
*Xin-Xiou-Ben-Cao *	659	850	635	128	87			
*Zheng-Lei-Ben-Cao *	1082	1,746	1,151	342	253		>3,000	
*Ben-Cao-Gang-Mu *	1596	1,892	1,094	443	161		11,096	194
*Znong-Yao-Da-Ci-Dian *	1977	5,767	4,773	740	82	172		
*Zhong-Hua-Ben-Cao *	1999	8,980	7,815	1,051	114			
Chinese Pharmacopoeia	2010	2,165	680	36	18	1,384		47

**Table 2:** Ethnic materia medica (EMM) in China.

Ethnic group	EMMs
*Han *	11,146
*Tibetan *	1,085
*Miao *	718
*Dai *	707
*Yi *	564
*Li-Su *	494
*Zhuang *	473
*Mongolian *	397
*Wa *	332
*Tu-Jia *	330
*Ha-Ni *	302
*De-Ang *	272
*A-Chang *	263
*Ji-Nuo *	250
*Du-Long *	165
*She *	161
*Mu-Lao *	152
*La-Hu *	151
*Uighur *	143
*Shui *	129
*Korean *	121
*Na-Xi *	103
*Bai *	90
*Mao-Nan *	75
*Pu-Mi *	49
*Bu-Lang *	44
*Bu-Yi *	32
*Beng-Long *	28
*Jing *	20
*Ge-Lao *	18
*Daur *	14
*Kazak *	14
*O-Lun-Chun *	12
*Hui *	11
*Manchu *	9
*Li *	9
*Yu-Gu *	5
*Gao-Shan *	4
*Tajik *	1
*Russian *	1
*Nu-Jiang *	1
*Wei-Xi *	1

Total	18,891

Data from “scientific database of China plant species. http://apps.searo.who.int/PDS_DOC”.
